# Identification of Neuropeptides as Potential Crosstalks Linking Down Syndrome and Periodontitis Revealed by Transcriptomic Analyses

**DOI:** 10.1155/2021/7331821

**Published:** 2021-09-10

**Authors:** Yue Chen, Xiaofei Yu, Jia Kong

**Affiliations:** ^1^Department of Pediatrics, Shanghai East Hospital, Tongji University School of Medicine, Shanghai 200120, China; ^2^Department of Medical Oncology, Shanghai Pulmonary Hospital, Thoracic Cancer Institute, Tongji University School of Medicine, Shanghai 200433, China; ^3^Department of Anesthesiology, Shanghai East Hospital, Tongji University School of Medicine, Shanghai 200120, China

## Abstract

**Background:**

This bioinformatics study was aimed to investigate the relationship between periodontitis (PD) and Down Syndrome (DS) regarding potential crosstalk genes, related neuropeptides, and biological processes.

**Methods:**

Data for PD (GSE23586, GSE10334 and GSE16134) and DS (GSE35665) were downloaded from NCBI Gene Expression Omnibus (GEO). Following normalization and merging of PD data, differential expression analysis was performed (*p* value < 0.05 and ∣log FC | ≥0.5). The common deregulated genes between PD and DS were considered as crosstalk genes. The significantly differentially expressed genes were used to construct the coexpression network and to further identify coexpression gene modules. To acquire the significant modules, the significant expression level of genes in the module was used to analyze the enrichment of genes in each module. Neuropeptides were assessed from NeuroPedia database. Neuropeptide genes and crosstalk genes were merged and mapped into PPI network, and the correlation coefficient (Spearman) was determined for the crosstalk genes.

**Results:**

138 crosstalk genes were predicted. According to the functional enrichment analysis, these genes significantly regulated different biological processes and pathways. In enrichment analysis, the significant module of DS was pink module, and turquoise module was significant in PD. Four common crosstalk genes were acquired, i.e., CD19, FCRL5, FCRLA, and HLA-DOB. In the complex network, INS and IGF2 interacted with CASP3 and TP53, which commonly regulated the MAPK signaling pathway. Moreover, the results showed that TP53 interacted with IGF2 and INS inducing the dysregulation of PI3K-Akt signaling pathway. UBL was positively correlated with crosstalk genes in both diseases. LEP was revealed to be both a neuropeptide and crosstalk gene and was positively correlated with other crosstalk genes.

**Conclusion:**

Different crosstalk genes, related neuropeptides, and biological pathways and processes were revealed between PD and DS, which can serve as a theoretical basis for future research.

## 1. Background

Periodontitis is an inflammatory disease of the tooth surrounding periodontal tissues, including marginal gingiva, periodontal ligament, and alveolar bone [1]. This primarily biofilm-associated multifactorial disease leads to a destruction of the respective soft and hard tissues, finally resulting in tooth loss [1]. Beside of the dysbiosis of oral microbiota, several general factors can influence the development and progression of periodontal diseases; thereby, systemic diseases can play a key role, and thus, periodontal manifestations of systemic diseases are defined in the recent classification of periodontal diseases [2]. Among others, Down Syndrome (DS) is included as a disease associated with immunologic disorders, which has a large influence on periodontal bone loss due to affected periodontal inflammation [2]. Thereby, patients with DS often suffer from an early onset of aggressive periodontitis, leading to tooth loss at a young age [3, 4]. This might be caused by several reasons. First, the altered microbiological composition of the oral cavity has been reported, whereby both, well-known potential periodontal pathogenic bacteria, e.g., *Porphyromonas gingivalis* or *Fusobacterium nucleatum* alongside with newly proposed taxa, e.g., *Filifactor*, *Fretibacterium*, or *Desulfobulbus* genera were found [5, 6]. This has even been reported in children aged 3-7 years [7]. Second, a different expression of genes in patients with DS can be related to periodontal inflammation, including inflammation-related genes [8], interferon-alpha and interferon-gamma related genes [9], or interleukin-10 signaling pathway genes [10]. Additionally, several immunological features like the altered relationship between MMP-8 and TIMP-2 [11] or oxidative burst activity of peripheral monocytes [12] have been reported. Moreover, the effect of co-factors, e.g., obesity might have an additional effect on periodontal disease severity and progression [13].

Altogether, the relationship between periodontitis and DS appears plausible, but complex. Thereby, a recent systematic review article stated that most of studies regarding associations between periodontitis and DS had methodological problems, making further research in the field necessary [14]. Therefore, this current study was aimed in assessing the crosstalk between periodontitis and DS by applying bioinformatics analysis. The topic should be examined from the perspective of potential crosstalk genes and examining different related neuropeptides in the interlink between the two diseases. It was hypothesized that there would be a crosstalk between periodontitis and DS, in which neuropeptides play a potential role.

## 2. Materials and Methods

### 2.1. Datasets

First, data for periodontitis (PD) and DS were downloaded from NCBI Gene Expression Omnibus (GEO). Thereby, the peripheral blood monocytes (PBM) samples within three datasets of PD (GSE23586, GSE10334, and GSE16134) and one dataset of DS (GSE35665) were acquired (Table 1).

For the PD and DS datasets, probe id to gene symbol was mapped based on GPL 570 and GPL5175, respectively. For the same genes, the average values of samples were calculated as the unique value. If the expression value for some genes was zero and the sample counts of this gene in case samples or control samples was more than half of the total number of samples, these genes were deleted. Subsequently, the gene expression value was normalized with the scale method of R project.

### 2.2. Differential Expression Analysis

For the PD, the common genes in GSE10334, GSE16134, and GSE23586 were acquired. These datasets were merged with ComBat package of R project. Then, the gene expression profile for the common genes was extracted. In order to reduce the differences between batches of samples when combined, the Combat method of “sva” package in R project was applied to make batch correction of the combined data. Differential expression analysis was performed for PD and DS with the “limma” package of R project. The genes with *p* value <0.05 and ∣log FC | ≥0.5 were considered as the differentially expressed genes (DEG). Meanwhile, the function of GO and KEGG pathway was analyzed with clusterProfiler of R project and the function with *p* value < 0.05 was significant.

### 2.3. Prediction of Crosstalk Genes

To predict the cross function between PD and DS, the DEGs of PD and DS were firstly intersected, and the common DEGs were considered as crosstalk genes. To expand the potential crosstalk genes, the PD- or DS-related genes were downloaded from the DisGeNET v6.0 (http://www.disgenet.org/). Finally, the known disease-related genes and the common DEGs between PD and DS were handled as the potential crosstalk genes.

### 2.4. Crosstalk Genes in the Weighted Gene Coexpression Network

The genes with *p* value < 0.05 in the differential expression analysis for PD and DS were selected, following extraction of the gene expression value, whereby the combined datasets of PD were used. The coexpression networks were constructed for PD and DS, respectively. The “wgcna” package of R program was used to construct the coexpression network and to further identify coexpression gene modules. WGCNA analysis was performed with the following steps: First, an unsupervised coexpression relationship was initially built based on the adjacency matrix of connection strengths by using Pearson's correlation coefficients for gene pairs. The power *β* was calculated, using the “pickSoftThreshold” function. The arguments (corFnc = ^“^bicor^”^, corOptions = list [maxPOutliers = 0.1], network type = ^“^signed^”^, power =  ^“^*β*^”^) were chosen to meet the need of scale-free topology property of the coexpression network. Based on the scale-free topology criterion, the optimum power *β* was selected to amplify the strong connections between genes and penalize the weaker connections.

The hybrid dynamic tree cutting method was used to cut branches and cluster coexpression modules in PD (cutHeight = 0.94, minSize = 20) and DS (cutHeight = 0.93, minSize = 20). To acquire the significant modules, the significant expression level of genes in the module was used to analyze the enrichment of genes in each module. Subsequently, the crosstalk genes from the significant modules of PD and DS were extracted. Afterwards, the functional enrichment analysis was performed to identify the biological processes and KEGG pathways with the clueGO of Cytoscape software. The function with *p* value < 0.05 was significant.

### 2.5. Crosstalk and Neuropeptides

In order to explore the role of crosstalk genes in the biological network, the known PPI relationship from 7 databases was downloaded, including HPRD (http://www.hprd.org/index_html), BIOGRID (http://thebiogrid.org/), DIP (http://dip.doe-mbi.ucla.edu/dip/M ain.cgi), MINT (http://mint.bio.uniroma2.it/mint/Welcome.do), menthe (http://mentha.uniroma2.it/index.php), PINA (http://cbg.garvan.unsw.edu.au/pina/), InnateDB (http://www.innatedb.com/), and Instruct (http://instruct.yulab.org/index.html). The PPI relationships were extracted for crosstalk genes, and the PPI network of crosstalk genes was constructed with Cytoscape software.

The human neuropeptides were assessed from NeuroPedia (http://proteomics.ucsd.edu/Software/NeuroPedia/). To explore the relationship between crosstalk genes and neuropeptides, neuropeptide genes and crosstalk genes were merged and mapped into the PPI network. Moreover, the pathways regulated by both, crosstalk genes and neuropeptides, were assessed. Finally, the PPI and pathway-gene relationship was integrated to build a functional complex network for crosstalk genes and neuropeptides.

In addition, the role of TF in the regulation between crosstalk genes and neuropeptides was analyzed. First, the TF regulated genes were downloaded from TRRUST (https://www.grnpedia.org/trrust), cGRNB (https://www.scbit.org/cgrnb), HTRIdb (http://www.lbbc.ibb.unesp.br/htri/), ORTI (http://orti.sydney.edu.au/about.html), and TRANSFAC (http://gene-regulation.com/pub/databases.html). Subsequently, the TF-target relationships for crosstalk genes and neuropeptides were assessed in the complex network, and the TF-target network of crosstalk genes and neuropeptides was constructed using the Cytoscape software. To further explore the role of neuropeptides and crosstalk genes, the gene expression value of neuropeptides and crosstalk genes was extracted in the disease samples of DS and PD to calculate the correlation coefficient (Spearman).

## 3. Results

### 3.1. Identification of DEGs Dysregulated in PD and DS

**Figure 1:**
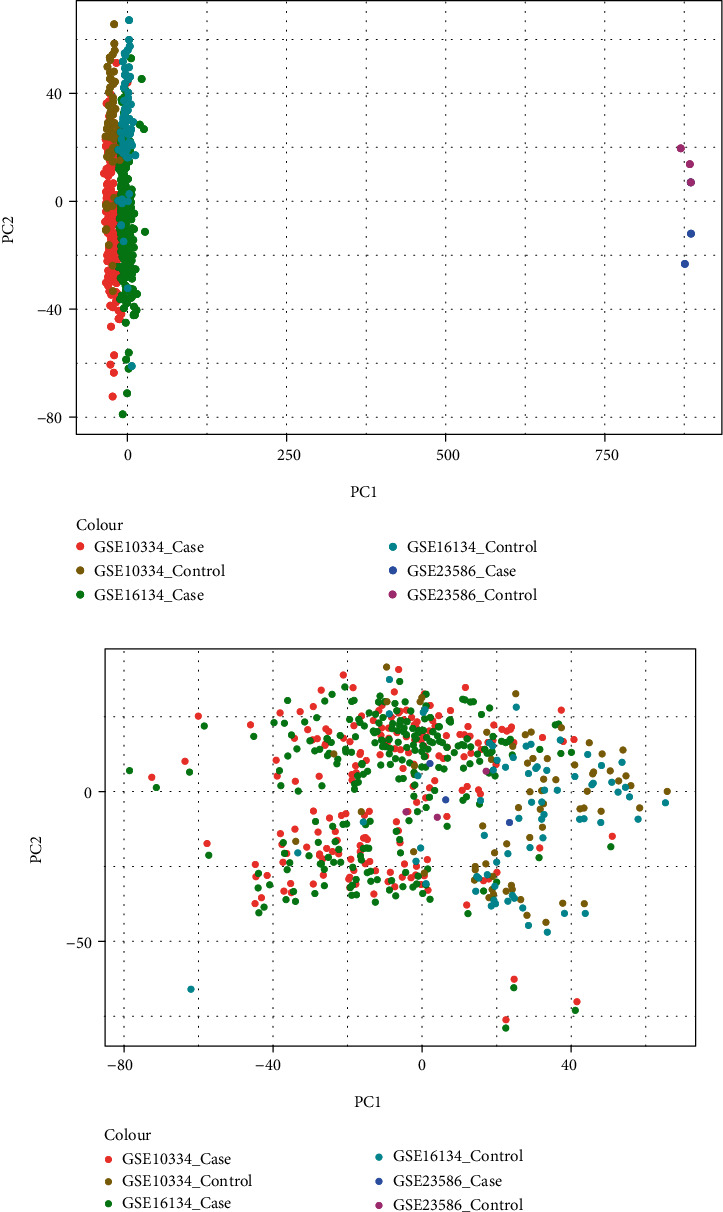
The differential expressed gene analysis. (a, b) PCA analysis results of three periodontitis data sets before and after batch correction.

**Figure 2:**
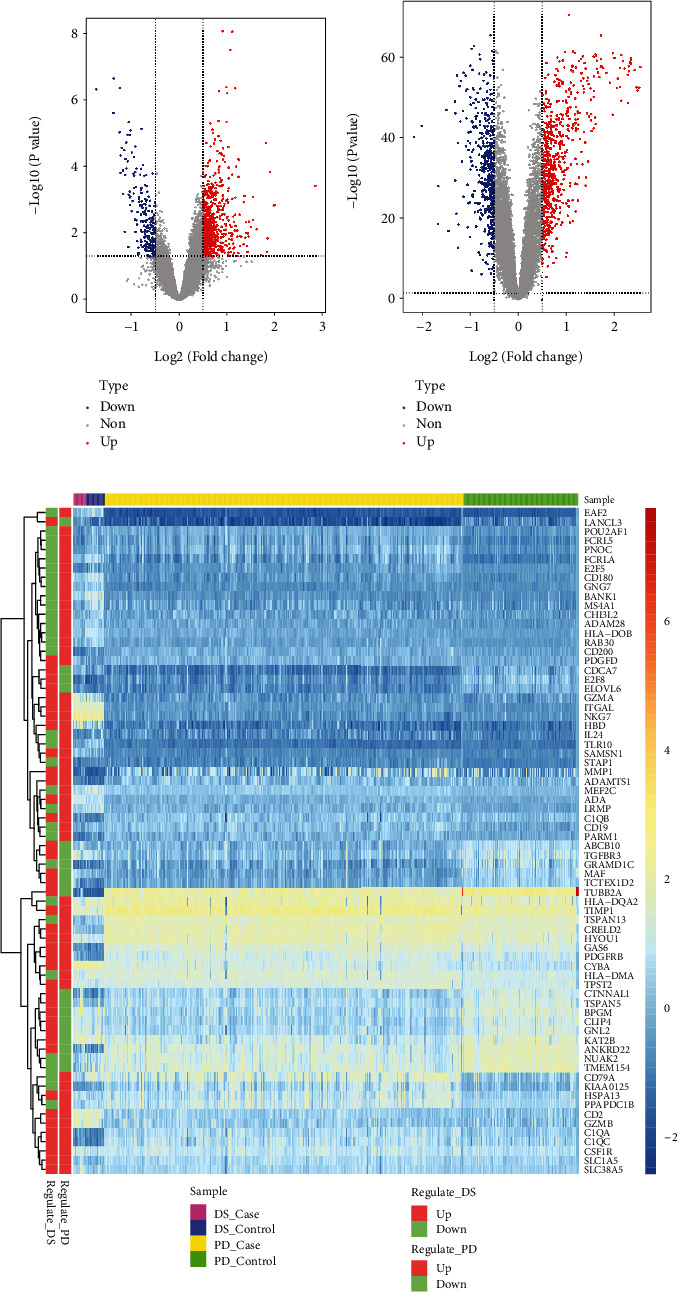
The differential expressed gene analysis. (a, b) The Volcano plot for the DEG of DS and PD. (c) The heat map of common DEGs between DS and PD.

It was found that there were differences among GSE10334, GSE16134, and GSE23586 before batch correction (Figure 1(a)), while the differences among the samples in the merged data after correction had significantly decreased (Figure 1(b)). 1125 DEGs of PD (Figure 2(b)) and 897 DEGs of DS (Figure 2(a)) were acquired. Moreover, 72 common DEGs between PD and DS were confirmed as the potential crosstalk genes (Figure 2(c)).

**Figure 3:**
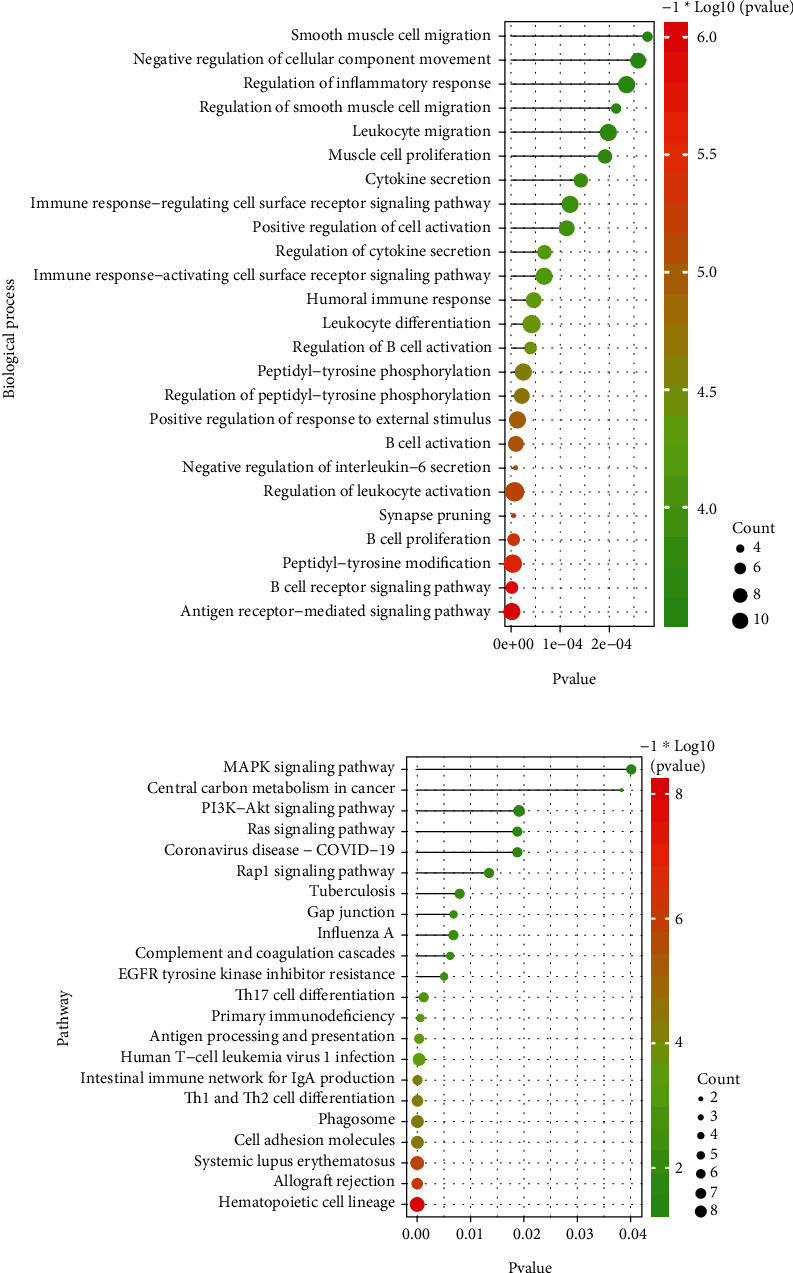
The significant functions of common DEGs between DS and PD. (a) The significant biological process and (b) significant pathways regulated by common DEGs.

With the “clusterProfiler” packages of R project, the results showed that the 72 common DEGs significantly regulated different biological and immunological pathways, e.g., antigen receptor-mediated signaling pathway and hematopoietic cell lineage (Figures 3(a) and 3(b)).

### 3.2. The Crosstalk Gene Prediction

**Figure 4:**
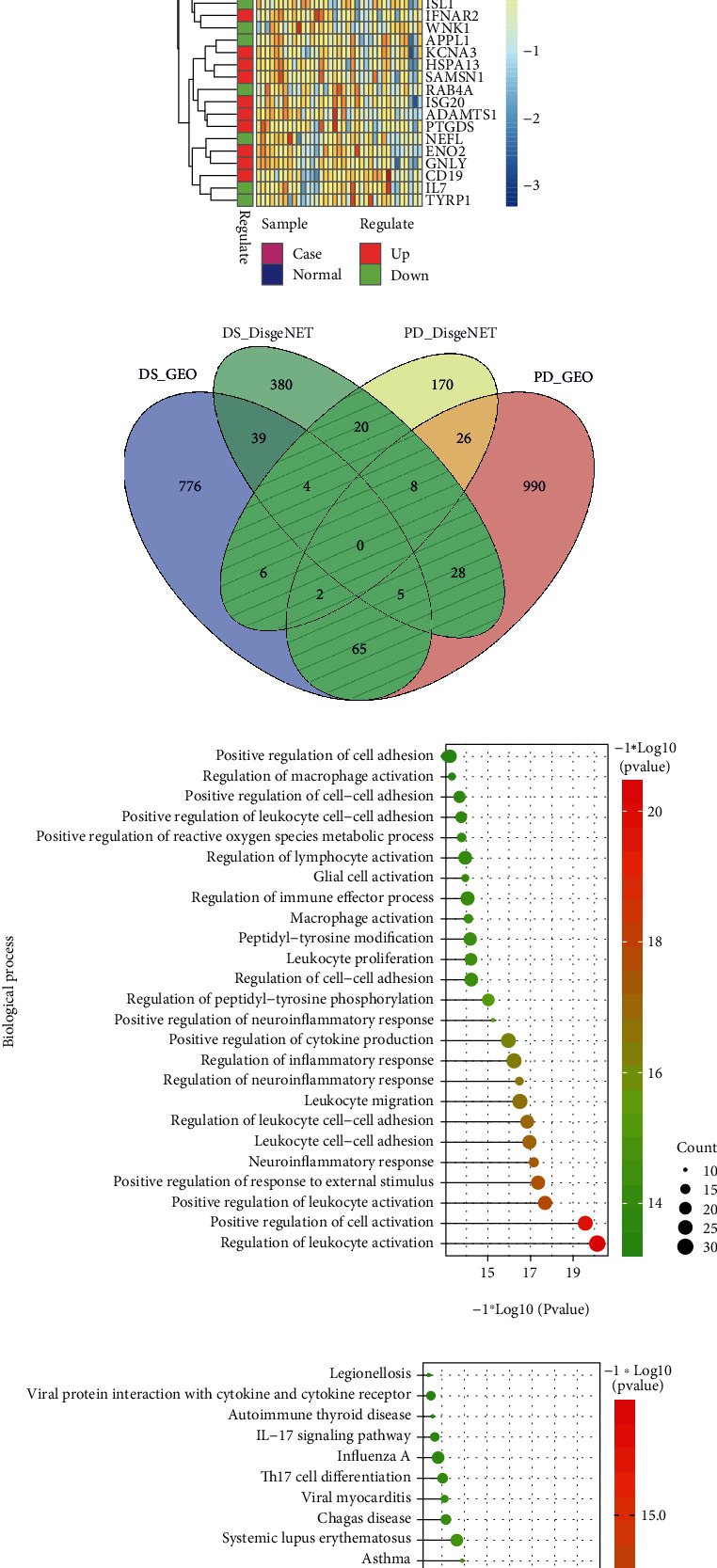
(a) Heat map of gene expression profiles of known PD genes in DS samples. (b) Heat map of gene expression profiles of known DS genes in PD samples. (c) Venn diagram for crosstalk genes. (d, e) Top 25 significant biological processes and pathways.

The expression of PD-related genes in DS samples is shown in Figure 4(a), and the gene expression of DS-related genes in PD samples is given in Figure 4B. Furthermore, any DS gene set and any PD gene set of the four gene sets (known PD-related genes and DEGs, known DS-related genes and DEGs) were combined to obtain their intersection genes as the final crosstalk gene set (Figure 4(c)). Finally, 138 crosstalk genes were predicted. According to the functional enrichment analysis, the 138 crosstalk genes significantly regulated different biological processes and pathways, which are presented in Figures 4(d) and 4(e).

### 3.3. Coexpression Modules to Screen the Potential Crosstalk Gene

**Figure 5:**
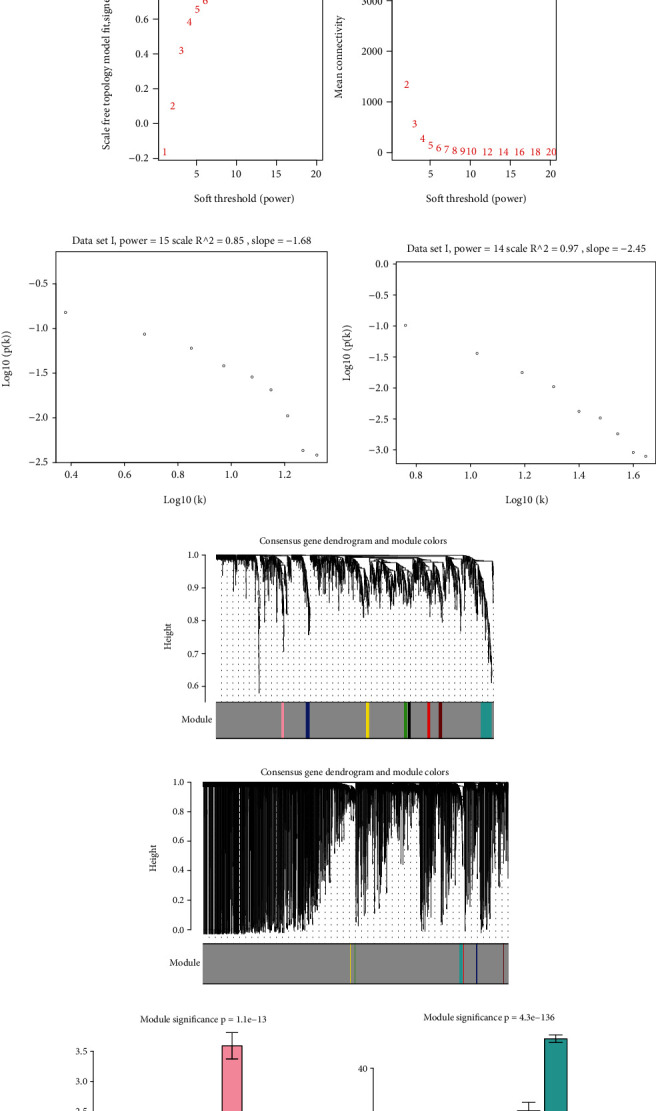
Identification of interesting modules in the DS and PD. (a, b) Scale-free ft index for different powers (*β*) of DS and PD. (c, d) Log-log plot of the whole-network connectivity distribution showing the quality of the relationship between connectivity (k) and P(K) for DS and PD. The *x*-axis shows the logarithm of whole network connectivity, and *y*-axis shows the logarithm of the corresponding frequency distribution. (e, f) Coexpressed modules obtained by average linkage hierarchical clustering of DS and PD. The color row underneath the dendrogram shows the module assignment determined by the Dynamic Tree Cut. (g, h) Bar plot of mean gene significance across modules. The height of the bars equals the mean value of -log10 (*p* value) across the observations with a given level of Module. By default, the barplot also shows plus/minus one standard error. The function also outputs the *p* value of a Kruskal Wallis test.

To construct the WGCNA module of PD and DS, the results showed that *β* = 15 was considered to obtain scale-free topology by the fit index greater than 0.85 for DS (Figure 5(a)), while *β* = 14 was suitable by the fit index greater than 0.85 for PD (Figure 5(b)). Using the scaleFreePlot of WGCNA to plot a log-log plot of a histogram of the given connectivity, and fit a linear model plus optionally a truncated exponential model, the *R*^2^ of the fit can be considered an index of the scale freedom of the network topology for DS and PD (Figures 5(c) and 5(c)). Finally, the acquired modules of DS and PD are shown in Figures 5(e) and 5(f), respectively. Subsequently, the differently expressed level was enriched into each module. From the enrichment analysis, it was found that the significant module of DS (Figure 5(g)) was the pink module, and the turquoise module was significant in PD (Figure 5(h)).

**Figure 6:**
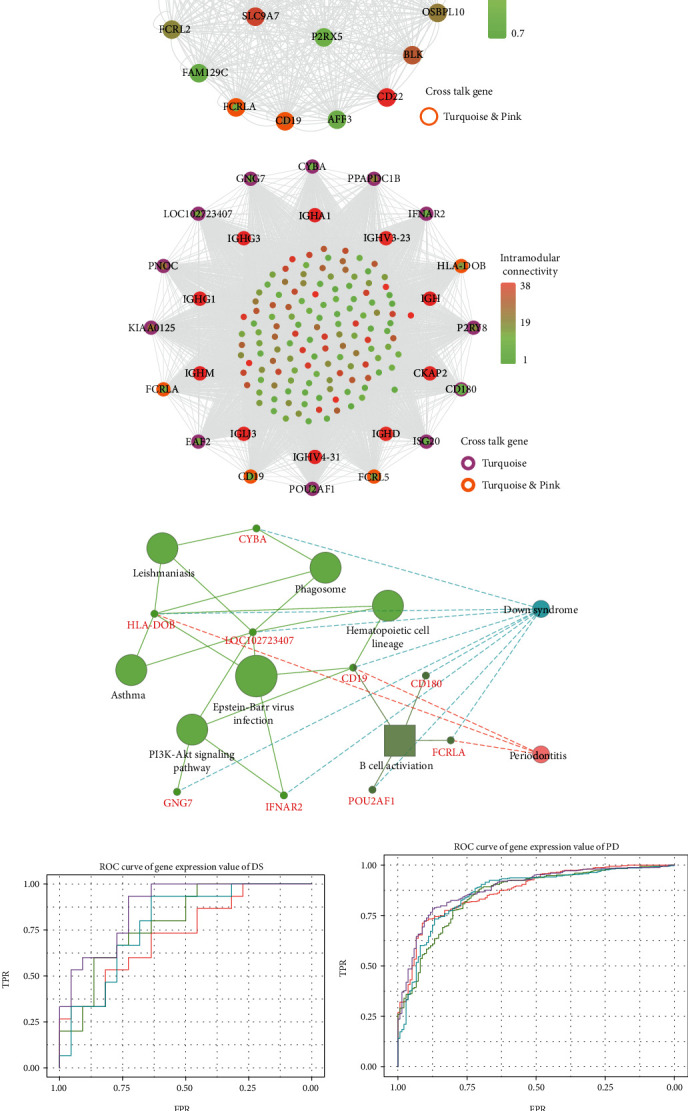
(a, b) PPI network for significant modules of DS and PD. The color of the midpoint in the coexpression network represents the difference in intramodular connectivity scores between a gene and other genes. Due to the large number of genes in the turquoise module for PD, we show the top 10 genes in the intramodular connectivity score and the crosstalk gene in the module. The crosstalk gene was trapped in different colored circles in different modules. (c) The function enrichment for the crosstalk genes in the significant module of DS and PD. (d, e) The prediction of common crosstalk gene in the significant module of DS and PD.

For the interaction of genes in module, the constructed PPI network for DS and PD is shown in Figures 6(a) and 6(b), which marked the crosstalk genes in each significant module. In the significant modules of PD and DS, 16 crosstalk genes and 4 common crosstalk genes were acquired (CD19, FCRL5, FCRLA, HLA-DOB). Subsequently, it was found that the 16 crosstalk genes significantly regulated the B-cell activation, Phagosome, PI3K-Akt signaling pathway, and Hematopoietic cell lineage (Figure 6(c)). Furthermore, the results of ROC analysis showed that the prediction accuracy of HLA−DOB in both, DS and PD, was higher than for the other three genes (Figures 6(d) and 6(e)).

### 3.4. Relationship between Crosstalk Genes and Neuropeptides

**Figure 7:**
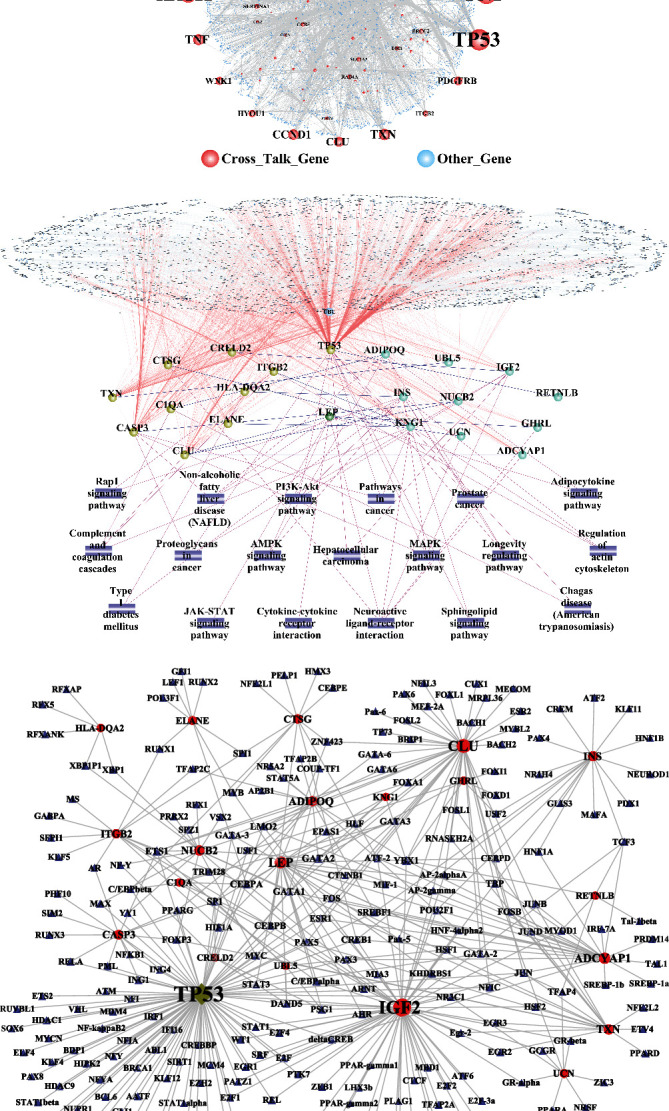
(a) The PPI network of crosstalk gene. The node size indicates the degree. The larger node showed that the degree of genes was higher than other genes. (b) The pathway-gene interaction network between crosstalk gene and neuropeptide. (c) TF-crosstalk/neuropeptide gene regulation network. The blue triangular node refers to the TF gene. TP53 is both of crosstalk gene and TF which marked the deep yellow diamond.

Figure 7(a) shows the constructed PPI network of crosstalk genes, including 3784 nodes and 5972 edges. The topological characteristics of the top 30 nodes ranked in the descending order of degree are displayed in Table 2. From the PPI network of crosstalk genes, the results showed that TP53 and FN1 played important roles in the whole biological network.

To explore the relationship between crosstalk genes and neuropeptides, the neuropeptide genes and 138 crosstalk genes were merged. Subsequently, the merged genes were mapped into the PPI network. Besides, the pathways regulated by both of crosstalk genes and neuropeptides were assessed. Finally, PPI and pathway-gene relationships were integrated to build a functional complex network for crosstalk genes and neuropeptides (Figure 7(b)). In the complex network, there were 10 neuropeptide genes, which interacted with crosstalk genes. INS and IGF2 interacted with CASP3 and TP53, which commonly regulated the MAPK signaling pathway. Moreover, the results showed that TP53 interacted with IGF2 and INS to induce the dysregulation of the PI3K-Akt signaling pathway.

To explore the role of TF in the regulation between crosstalk genes and neuropeptides, the TF-target relationships for 11 crosstalk genes and 10 neuropeptides in the complex network were extracted, and the TF-target network of crosstalk genes and neuropeptides was constructed with Cytoscape software (Figure 7(c)). The results showed that TP53 was regulated by more TF than other crosstalk genes. Meanwhile, TP53 was also TF, which regulated other genes. IGF2 and CLU were regulated by more TF than other neuropeptides.

### 3.5. Correlation between Neuropeptides and Crosstalk Genes in PD and DS

**Figure 8:**
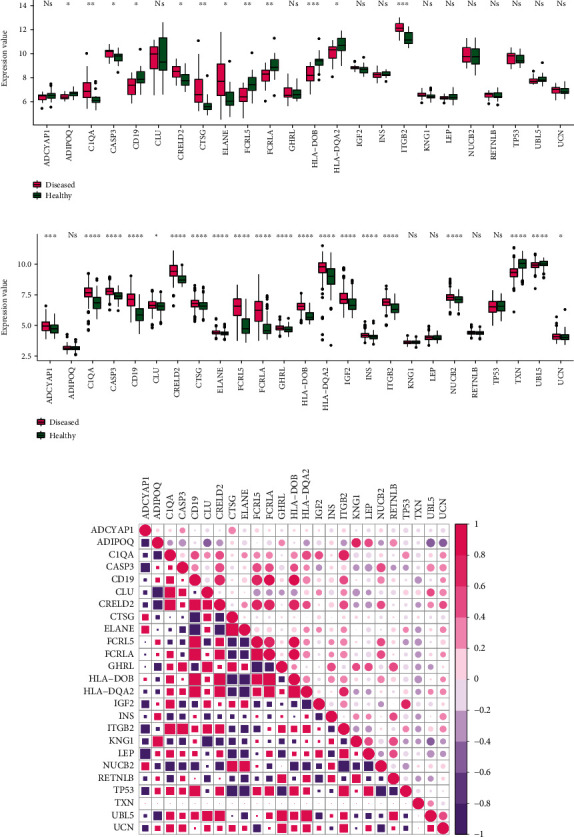
(a, b) Gene expression level of neuropeptides and crosstalk genes in DS and PD. The gene with red mark is neuropeptides. LEP is both neuropeptide and crosstalk gene marked with “^∗^”. (c) The correlation coefficient of neuropeptides and crosstalk genes in DS and PD. The gene marked with “^∗^” were neuropeptides. LEP is both neuropeptide and crosstalk gene marked with “^∗∗^”.

The 11 crosstalk genes and 10 neuropeptides were merged in the complex network, and the four common crosstalk genes in the significant module in PD and DS were assessed. Finally, 25 genes were further analyzed. First, the gene expression values of 25 genes in the disease samples of DS and PD were extracted (Figure 8(a) and 8(b)). The results showed that the gene expression level of neuropeptides in DS was higher than in PD. HLA-DOB and HLA-DQA2 were crosstalk genes in DS and PD.

The correlation coefficient of neuropeptides and crosstalk genes in DS and PD, analyzed with Spearman Correlation Coefficient, is shown in Figure 8(c). Thereby, the correlation of neuropeptide genes with crosstalk genes was lower in PD than in DS. UBL was positively correlated with crosstalk genes in both DS and PD and regulated the Neuroactive ligand-receptor interaction. LEP is both a neuropeptide and crosstalk gene. The results showed that LEP was negatively correlated with most of other neuropeptide genes and positively correlated with crosstalk genes in DS and PD.

## 4. Discussion

### 4.1. Main Results

Four common crosstalk genes between periodontitis and DS were acquired, i.e., CD19, FCRL5, FCRLA, and HLA-DOB, of which the latter had the highest prediction accuracy. B-cell activation, Phagosome, PI3K-Akt signaling pathway, and Hematopoietic cell lineage were identified as the most relevant pathways. The neuropeptides INS and IGF2 interacted with CASP3 and TP53, potentially affecting the MAPK signaling pathway, and TP53 interacted with IGF2 and INS to induce the dysregulation of the PI3K-Akt signaling pathway. LEP was found to be both, neuropeptide and crosstalk gene.

### 4.2. Comparison with Literature and Interpretation

This is the first bioinformatics study, which investigated the potential crosstalk between DS and periodontitis. A genetic crosstalk between periodontitis and DS has been repeatedly discussed in literature [8–10, 15, 16]. This has been evaluated in the current study, whereby the hypothesis of a potential crosstalk between these two diseases was confirmed. Thereby, a recent case-control study identified single nucleotide polymorphisms (SNP) to be significantly associated to periodontal disease in DS individuals, underlining the relevance of shared genetic mechanisms [15]. It has also been found in that clinical study that different metabolic pathways, including the PI3K-Akt signaling pathway, would play an important role in this context [15]. This was also a result of the current study, where crosstalk gene TP53 and interacting neuropeptides IGF2 and INS were related to the PI3K-Akt signaling pathway. It was shown that the PI3K-Akt signaling pathway would be related to virulence factors (gingipains) of *Porphyromonas gingivalis* (*P.g.*), a common potential periodontal pathogen [17]. An altered microbiological composition of the oral cavity from patients with DS has been reported in clinical studies [5, 6]. A comparative clinical trial found increased presence of *P.g.* in DS patients with periodontitis [18]. The role of INS can be supported by the fact that supernatants of potential periodontal pathogens, i.e., *P.g.* and *Tanerella forsythia* were found to potentially stimulate insulin secretion in pancreatic *β*-cells, promoting the induction of proinflammatory molecules by activating the PI3K/AKT signaling pathway [19]. IGF2, which was also relevant in the current analysis, was found to be involved in different biological processes in periodontal ligament cells [20]. Moreover, TP53 has an influence on cell proliferation and differentiation of dental stem cells [21]. Accordingly, this revealed relationship between neuropeptides, crosstalk gene, and pathway might be associated to the action of common potential periodontal pathogens and related immunological and metabolical reactions.

LEP was found to be both, a crosstalk gene as well as a relevant neuropeptide. Leptin is known as an important player in metabolic control, alongside with reproduction and neuroendocrine signaling [22]. As a relationship between metabolic syndrome, obesity, and diabetes with periodontal disease is well documented in literature, the role of LEP has been repeatedly investigated in this context [23–25]. While one case-control study did not find an association between LEP and periodontitis [23], another study found positive correlations between LEP and worse periodontal parameters [24]. Considering that, the findings of a prospective observational study, which indicate an association between obesity and periodontitis in individuals with DS, appear of particular interest [13].

Few previous studies revealed genes, which were differentially expressed in patients with DS in the case of periodontitis [8–10, 15]. Within these studies, none out of the four crosstalk genes revealed in the current study (CD19, FCRL5, FCRLA, and HLA-DOB) was found, too. However, while three studies only examined selected genes, excluding the crosstalk genes from the current study [8–10], the other available study examined salivary samples [15], making the comparability with the current study questionable. Nonetheless, there is a certain plausibility for the clinical relevance of the detected crosstalk genes in the current study. HLA-DOB has been demonstrated to be related to autoimmunity in celiac disease and ankylosing spondylitis [26, 27]. Because periodontitis is related to autoinflammatory diseases [28], the relevance of this crosstalk gene appears reasonable. The potential relevance of HLA class antigens in DS patients with periodontitis has already been highlighted in a comparative study [16]. Thus, an increase in (auto)inflammatory activity in patients with DS could be a further explanation for their high periodontal burden. This could be supported by the role of CD19 as crosstalk gene; CD19 positive breg cells were found to be associated with periodontal disease progression and were reported to be a possible link between periodontal and systemic inflammation [29]. Similarly, CD19 was already examined in context of premature aging of the immune system and proinflammatory profile of DS [30].

Taken together, three potential ways of an interlink between periodontitis and DS could be assumed based on the current study's findings: (i) patients with DS have an altered microbiological environment in the oral cavity. Increased level of potentially periodontal pathogens and their virulence factors might affect an interaction of TP53 with IGF2 and INS to induce the dysregulation of the PI3K-Akt signaling pathway. (ii) Obesity, metabolic syndrome, and LEP could play a role in the relationship between periodontitis and DS. (iii) A proinflammatory or autoinflammatory profile of patients with DS induced an early on setting severe periodontitis. Probably, a combination of these mechanisms could exist; however, this remains speculative.

### 4.3. Strengths and Limitations

This is the first bioinformatics study on the interlink between periodontitis and DS. The combination of crosstalk genes, neuropeptides, and pathways was very comprehensive and is up until now unique in literature. Other working groups [31, 32] have already successfully applied similar methodology previously to reveal the link between periodontal and systemic diseases. However, several limitations exist. The sample size of DS patients was quite small and far smaller than for periodontitis samples. Moreover, the groups were very heterogeneous regarding age, gender, and comorbidities. Thereby, different patients with DS or periodontitis were analyzed, respectively. Only peripheral blood samples were considered, which is a further limitation. The main point that needs to be mentioned here is that these findings need clinical validation to be confirmed. Besides these limitations, the current study's results can serve as a good theoretical basis for future research in the field.

## 5. Conclusion

Different crosstalk genes, related neuropeptides, biological pathways, and processes were revealed between periodontitis and DS. Thereby, the PI3K-Akt signaling pathway, leptin-associated metabolic processes, and a proinflammatory profile of the immune system might predict patients with DS to develop an early on setting, severe aggressive periodontitis. These approaches need further clinical validation to be confirmed.

## Figures and Tables

**Table 1:** The datasets for the current study.

Data set	Disease	Platform	Case samples	Control samples	Gene number
GSE10334	PD	GPL570	183	64	24441
GSE16134	PD	GPL570	241	69	24441
GSE23586	PD	GPL570	3	3	23518
GSE35665	DS	GPL5175	15	22	17300

**Table 2:** The topological characteristics of the top 30 nodes with the greatest degree in the PPI.

Name	Label	Degree	Average shortest path length	Betweenness centrality	Closeness centrality	Topological coefficient
TP53	Cross	1079	2.251789	0.420797	0.444091	0.002318
FN1	Cross	818	2.451365	0.305578	0.407936	0.003156
HSPD1	Cross	275	2.381129	0.095272	0.419969	0.005405
CASP3	Cross	196	2.800159	0.058752	0.357123	0.009409
KAT2B	Cross	194	2.632123	0.057222	0.379921	0.006785
TXN	Cross	132	2.612775	0.037883	0.382735	0.009874
TNF	Cross	129	2.909091	0.04546	0.34375	0.012626
TUBB2A	Cross	124	2.68036	0.035164	0.373084	0.009755
CCND1	Cross	121	2.916247	0.028949	0.342906	0.01551
CLU	Cross	116	2.814471	0.036914	0.355307	0.012297
TGFB1	Cross	105	2.953883	0.037888	0.338537	0.018972
PDGFRB	Cross	100	3.001855	0.029277	0.333127	0.029775
ENO2	Cross	98	2.971376	0.025504	0.336544	0.019222
APPL1	Cross	94	2.7066	0.033818	0.369467	0.012009
WNK1	Cross	92	3.028359	0.029407	0.330212	0.027433
HYOU1	Cross	80	2.986483	0.020004	0.334842	0.023633
UBC	Cross	72	2.118208	0.170684	0.472097	0.020807
APOE	Cross	70	2.999205	0.022849	0.333422	0.027381
ITGB2	Cross	62	2.994699	0.01851	0.333923	0.03201
TSPAN5	Cross	61	3.040021	0.027445	0.328945	0.020492
NOD2	Cross	54	3.06626	0.015464	0.32613	0.032764
SERPINA1	Cross	52	2.786112	0.016528	0.358923	0.021452
ERCC2	Cross	51	2.750066	0.013578	0.363628	0.02154
NEFL	Cross	49	2.994964	0.013643	0.333894	0.027498
RAB4A	Cross	49	2.749536	0.016342	0.363698	0.021511
CCR5	Cross	47	3.181818	0.011847	0.314286	0.032499
GHR	Cross	47	3.042937	0.009512	0.32863	0.043566
GZMB	Cross	45	2.9022	0.009937	0.344566	0.024898
SLC1A5	Cross	45	3.067851	0.01041	0.325961	0.047009
CTSG	Cross	41	2.967135	0.01082	0.337025	0.026546

## Data Availability

The data used to support the findings of this study are available from the corresponding author upon request.
